# Theoretical Determination of High-Energy Photon Attenuation and Recommended Protective Filler Contents for Flexible and Enhanced Dimensionally Stable Wood/NR and NR Composites

**DOI:** 10.3390/polym13060869

**Published:** 2021-03-11

**Authors:** Worawat Poltabtim, Donruedee Toyen, Kiadtisak Saenboonruang

**Affiliations:** 1 Department of Applied Radiation and Isotopes, Faculty of Science, Kasetsart University, Bangkok 10900, Thailand; wp.worawat@gmail.com; 2 Scientific Equipment and Research Division, Kasetsart University Research and Development Institute (KURDI), Kasetsart University, Bangkok 10900, Thailand; rdiddt@ku.ac.th; 3 Specialized Center of Rubber and Polymer Materials in Agriculture and Industry (RPM), Faculty of Science, Kasetsart University, Bangkok 10900, Thailand

**Keywords:** natural rubber, wood particles, Bi_2_O_3_, Bi_2_S_3_, Pb, photon, gamma, X-ray, shielding, XCOM

## Abstract

This work aimed to theoretically determine the high-energy-photon-shielding properties of flexible wood/natural rubber (NR) and NR composites containing photon protective fillers, namely Pb, Bi_2_O_3_, or Bi_2_S_3_, using XCOM. The properties investigated were the mass attenuation coefficient (*µ_m_*), linear attenuation coefficient (*µ*), and half value layer (*HVL*) of the composites, determined at varying photon energies of 0.001–5 MeV and varying filler contents of 0–1000 parts per hundred parts of rubber by weight (phr). The simulated results, which were in good agreement with previously reported experimental values (average difference was 5.3%), indicated that overall shielding properties increased with increasing filler contents but decreased with increasing incident photon energies. The results implied the potential of bismuth compounds, especially Bi_2_O_3_, to replace effective but highly toxic Pb as a safer high-energy-photon protective filler, evidenced by just a slight reduction in *µ_m_* values compared with Pb fillers at the same filler content and photon energy. Furthermore, the results suggested that the addition of 20 phr wood particles, primarily aimed to enhance the rigidity and dimensional stability of Pb/NR, Bi_2_O_3_/NR, and Bi_2_S_3_/NR composites, did not greatly reduce shielding abilities; hence, they could be used as dimensional reinforcers for NR composites. Lastly, this work also reported the optimum Pb, Bi_2_O_3_, or Bi_2_S_3_ contents in NR and wood/NR composites at photon energies of 0.1, 0.5, 1, and 5 MeV, with 316–624 phr of filler being the recommended contents, of which the values depended on filler type and photon energy of interest.

## 1. Introduction

High-energy photons, especially X-rays and gamma rays, are presently used in various applications, for instance, X-ray and gamma imaging for medical and industrial purposes [1,2,3], radiotherapy for cancer treatment [4], gamma irradiation on plants for mutation breeding [5], and X-ray fluorescence (XRF) for elemental analysis [6]. However, despite their benefits and potentials, excessive exposure to high-energy photons could fatally harm users and the general public [7], with the symptoms including skin burn, loss of appetite, nausea, vomiting, abdominal pain, fever, diarrhea, loss of hair, damage to bone marrow, cancer, and even death [8,9,10]. Herein, a radiation safety principle called “As Low As Reasonably Achievable” or “ALARA” must be strictly followed in all radiation- and nuclear-related facilities [11].

One of the three ALARA principles is the use of effective and appropriate radiation-shielding equipment that could sufficiently reduce radiation exposure on users to within occupational dose limits recommended by the International Commission on Radiological Protection (ICRP) [12]. However, as technologies relying on the use of high-energy photons are rapidly expanded, development in novel and better radiation-shielding equipment is constantly pursued with specific added-on properties such as flexibility, transparency, environmental friendliness, and self-healing ability. Examples of newly developed high-energy photon-shielding materials are self-healing gamma-shielding hydrogels from nano-bismuth oxide (nano-Bi_2_O_3_)/poly(vinyl) alcohol (PVA) [10], flexible ethylene propylene diene monomer (EPDM) and natural rubber (NR) composites containing metal oxides [13,14], biocompatible polyaniline reinforced with hybrid graphene oxide-iron tungsten nitride flakes [15], and environmental-friendly Bi_2_O_3_-filled concrete [16].

Among these materials used for the production of radiation-shielding equipment, NR has presented itself as one of the most interesting materials due to excellent tensile properties, high tear resistance, lower odor than synthetic rubber (SR), and waterproofing ability [17]. However, pristine NR is not readily the most effective shielding materials as NR mostly consists of light elements that have low interaction probabilities with high-energy photons. As a result, appropriate fillers must be incorporated into the NR matrix to increase the probability of interactions. Specifically for X-ray and gamma rays, it has been known and widely accepted that heavy metals and heavy-metal-containing compounds, especially involving lead (Pb), are generally used as economical and effective protective fillers due to their high atomic numbers (*Z*) and densities (*ρ*), which could increase interactions between materials and incident photons through the mechanisms of the photoelectric absorption, Compton scattering, and pair production that consequently result in better radiation attenuation [18,19]. In addition, Pb is also economically accessible such that low-cost shielding materials could be produced [20]. However, despite its advantages, Pb is highly toxic and hazardous to humans, animals, plants, and the environment as it could damage the brain and blood systems of living organisms, causing abdominal pain, constipation, headaches, irritability, and even death [21]. Therefore, alternative heavy-metal-containing compounds such as bismuth compounds, especially Bi_2_O_3_ and Bi_2_S_3_, which are considered safer for producers and users than Pb, has gained much attention from researchers [13,14]. For example, the report from Poltabtim et al. showed that EPDM composites containing 500 phr Bi_2_O_3_ had a linear attenuation coefficient (*µ*) of 24.3 ± 2.2 m^−1^ at a gamma energy of 1.25 MeV [13], which was comparable with NR composites containing the same Pb content that had a *µ* value of 26.0 m^−1^ [22]. This comparison implied the high potential of Bi_2_O_3_ and Bi_2_S_3_ to replace Pb as effective and safe protective fillers.

Although high contents of Bi_2_O_3_ and Bi_2_S_3_ in NR composites generally resulted in enhanced shielding properties, the overall mechanical properties of the composites could be inversely reduced, mostly due to their poor interfacial compatibility and the different contraction/expansion behaviors during curing and cooling processes between NR and Bi_2_O_3_ (Bi_2_S_3_), which possibly created voids between the surfaces of the two substances. Furthermore, high filler contents could also lead to agglomerations of particles from filler-filler interactions. Consequently, these effects lowered the overall strength and flexibility of the materials. Examples of the effects from high filler contents are a decrease in the tensile strength of Bi_2_O_3_/NR composites from ~13 MPa in a sample with 100-phr Bi_2_O_3_ to ~8 MPa and ~6 MPa for samples with 300-phr and 500-phr Bi_2_O_3_, respectively [13]. Thus, the effects of radiation protective fillers in improving shielding properties but also lowering the overall mechanical properties of the composites are two competing factors which researchers and product developers must pay close attention to in order to produce NR composites that are effective, safe, and usable for all the intended applications.

In addition to the filler contents that could affect the shielding and mechanical properties of NR composites, the self-solid-lubricant properties of most metal compounds added to NR could result in decreased crosslink densities, shortened lifetimes, and unpleasantly expanded dimensions of the NR composites after some periods of usage [23]. For instance, Ninyong et al. reported that the dimensions of 80-phr B_2_O_3_/NR composites, which were developed as flexible neutron shielding materials, increased by 44% after normal usage at room temperature for 150 days. Subsequently, this unfortunate behavior led to a significant reduction in the shielding abilities of the material (approximately 36%) due to the decrease in material thickness by 31% that resulted in lower photon interactions with the materials [24]. To enhance dimensional stability of the NR composites, fillers with high rigidity and roughness such as wood particles could be used by incorporating them into NR composites. This improvement in dimensional stability after the addition of wood particles was also confirmed by Ninyong et al., who showed that by adding 20 phr of wood particles into NR composites, the dimension of 80-phr B_2_O_3_/NR composites increased by just 14% after 150 days of usage, resulting in a decrease in the shielding abilities by 13% compared to the 36% drop in those without the addition of wood particles [24]. Clearly, the wood particles showed potential to be used as a dimensional reinforcer in Bi_2_O_3_/NR and Bi_2_S_3_/NR composites, especially in the case of high filler contents.

Consequently, the current work aimed to theoretically determine the high-energy photon shielding properties, based on the mass attenuation coefficient (*µ_m_*), linear attenuation coefficient (*µ*), and half value layer (*HVL*) of Pb/NR, Bi_2_O_3_/NR, and Bi_2_S_3_/NR composites with or without the addition of wood particles using XCOM provided by the National Institute of Standards and Technology (NIST) [25]. To fully understand effects of filler contents and photon energies on shielding properties, the Pb and Bi_2_O_3_ contents as well as the photon energy used in the simulation were varied in the ranges 0–1,000 phr and 0.001–5 MeV, respectively, and the obtained results were compared with previously reported experimental values with similar material preparations and testing procedures to ensure the correctness and the reliability of the simulated results. Furthermore, the investigation reported and discussed the recommended filler contents of Pb, Bi_2_O_3_, and Bi_2_S_3_ in wood/NR and NR composites at various gamma energies (0.1 MeV, 0.5 keV, 1 MeV, and 5 MeV), which were selected based on the determination of the least filler content that increased the *µ* value of the composites by less than 5% after the addition of 40 phr of the filler. This content optimization was carried out to minimize the negative effects on the mechanical properties of the composites from high filler contents while still offering relatively high shielding ability.

## 2. Simulation Setup and Determination of High-Energy Photon Shielding Properties

### 2.1. Simulation Setup

The web-based XCOM software provided by the National Institute of Standards and Technology (NIST), Gaithersburg, MD, USA, was used to determine the values of *µ_m_* in the wood/NR and NR composites containing Bi_2_O_3_, Bi_2_S_3_, or Pb particles (*µ_m_* represent the fraction of attenuated incident photons in a monoenergetic beam per unit mass) [25]. Originally, the XCOM software was developed to ease tedious and complicated numerical calculation of photon cross sections for compounds, especially at the absorption edges, which was usually carried out by finding weighted sums of individual cross sections for all of the atomic constituents. Furthermore, cross sections at energies immediately above and below all absorption edges, which have discontinuous photo-absorption cross sections that lead to complications in usual numerical calculation, are automatically included in XCOM. The photon cross section database used in this work was the NIST standard reference database 8 (XGAM), released in November 2010. The contents of the chemicals used for the wood/NR and NR composites were entered as a mixture, with the formulation of the composites shown in Table 1, and the photon energies were entered from 0.001–5 MeV. It should be noted that the values of *µ_m_* reported in this work were calculated from the total attenuation with the inclusion of coherent scattering [26].

### 2.2. Determination of µ and HVL

The values of *µ* and *HVL*, which represent the fraction of attenuated incident photons in a monoenergetic beam per unit thickness and the thickness of the materials required to attenuate 50% of incident photons, respectively, of the wood/NR and NR composites containing Bi_2_O_3_, Bi_2_S_3_, or Pb particles with contents varying from 0 to 1000 phr at photon energies of 0.001–5 MeV were determined from the obtained values of *µ_m_* (XCOM) using Equations (1) and (2), respectively:

(1) $$\mu =\mu_{m}\rho$$

(2) $$HVL=\frac{\ln\left(2\right)}{\mu }$$

where *ρ* is the density of the composites theoretically estimated using Equation (3):

(3) $$\rho =\frac{\sum_{i}c_{i}}{\sum_{i}\frac{c_{i}}{\rho_{i}}}$$

where *i* is the *i^th^* chemical in Table 1, *ρ_i_* is the density of the *i^th^* chemical in Table 1, and *c_i_* is the content of the *i^th^* chemical in Table 1 [13].

### 2.3. Determination of Recommended Bi_2_O_3_, Bi_2_S_3_, and Pb Contents

The recommended Bi_2_O_3_, Bi_2_S_3_, and Pb contents at photon energies of 0.1, 0.5, 1, and 5 MeV were determined by considering the percentage change in *µ_m_* (%*change*) after the addition of 40 phr of fillers and was calculated using Equation (4):

(4) $$\%\;change=\;\frac{\mu_{m,2}-\mu_{m,1}}{\mu_{m,1}}$$

where *µ_m_*_,1_ is the initial *µ_m_* of the composites and *µ_m,_*_2_ is the resulting *µ_m_* of the composites after the additional 40-phr Bi_2_O_3_, Bi_2_S_3_, or Pb.

To obtain the recommended contents for each filler type and photon energy, %*change* for all contents (0–1000 phr) were determined and the least contents that had %*change* less than 5% were selected as the recommended contents (the threshold of %*change*, which was 5% in this work, could be varied depending on users’ preferences). Furthermore, in order to determine recommended contents at any photon energies besides the reported values at 0.1, 0.5, 1, and 5 MeV, trendlines were also developed using equations in the form:

(5) $$f\left(x\right)=Ax^{B}$$

where *f(x)* is the threshold of %*change*, *x* is the recommended filler content, and *A* and *B* are mathematical constants determined using a trendline function available in Microsoft Excel. It should be noted that the objective of this determination was to minimize the effects of too-high filler contents on lowering the mechanical properties of the composites.

## 3. Results and Discussion

### 3.1. Mass Attenuation Coefficient (µ_m_)

**Figure 1:**
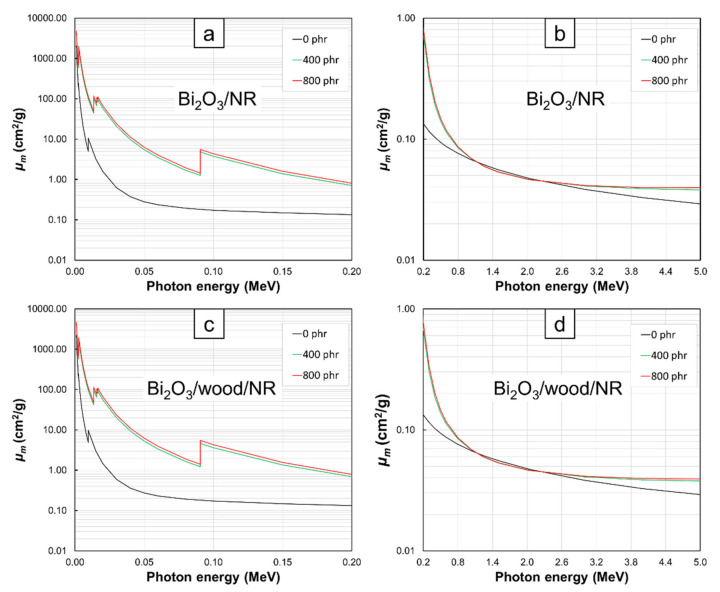
*µ_m_* values of (**a**,**b**) Bi_2_O_3_/NR and (**c**,**d**) Bi_2_O_3_/wood/NR composites with filler contents of 0, 400, and 800 phr, determined at photon energies of (**a**,**c**) 0.001–0.2 MeV and (**b**,**d**) 0.2–5 MeV using XCOM. Raw data are provided in the Supplementary Materials (Tables S1, S2, S3, S8, S9, and S10).

**Figure 2:**
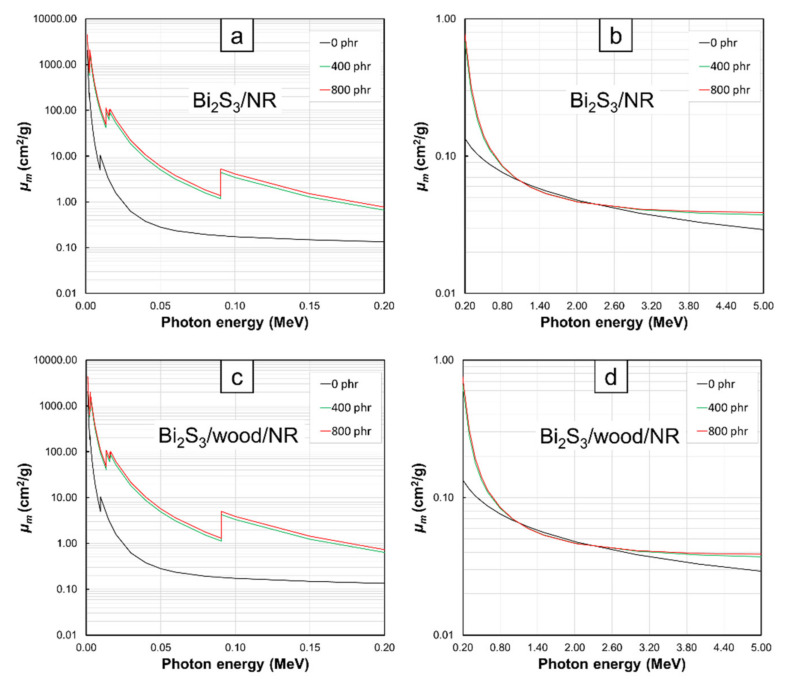
*µ_m_* values of (**a**,**b**) Bi_2_S_3_/NR and (**c**,**d**) Bi_2_S_3_/wood/NR composites with filler contents of 0, 400, and 800 phr, determined at photon energies of (**a**,**c**) 0.001–0.2 MeV and (**b**,**d**) 0.2–5 MeV using XCOM. Raw data are provided in the Supplementary Materials (Tables S1, S6, S7, S8, S13, and S14).

**Figure 3:**
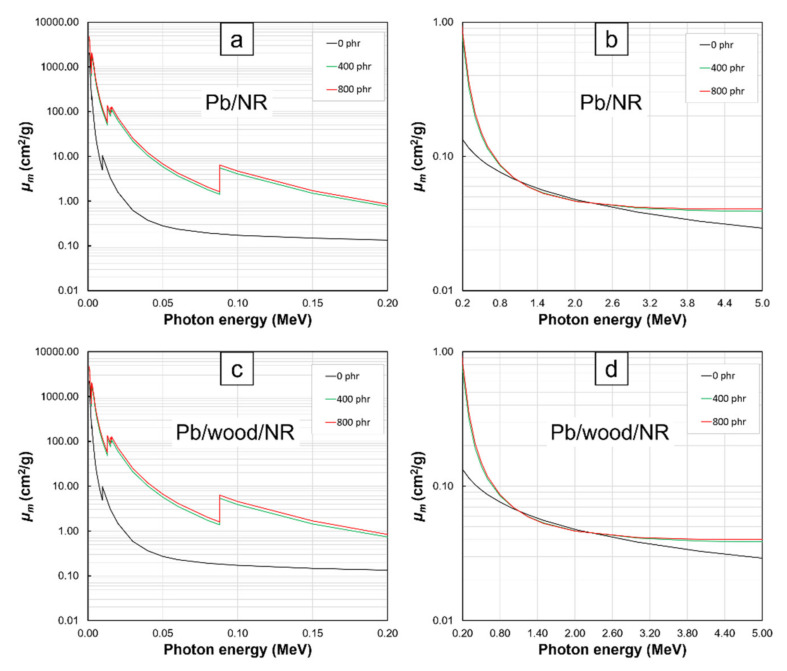
*µ_m_* values of (**a**,**b**) Pb/NR and (**c**,**d**) Pb/wood/NR composites with filler contents of 0, 400, and 800 phr, determined at photon energies of (**a**,**c**) 0.001–0.2 MeV and (**b**,**d**) 0.2–5 MeV using XCOM. Raw data are provided in the Supplementary Materials (Tables S1, S4, S5, S8, S11, and S12).

**Figure 4:**
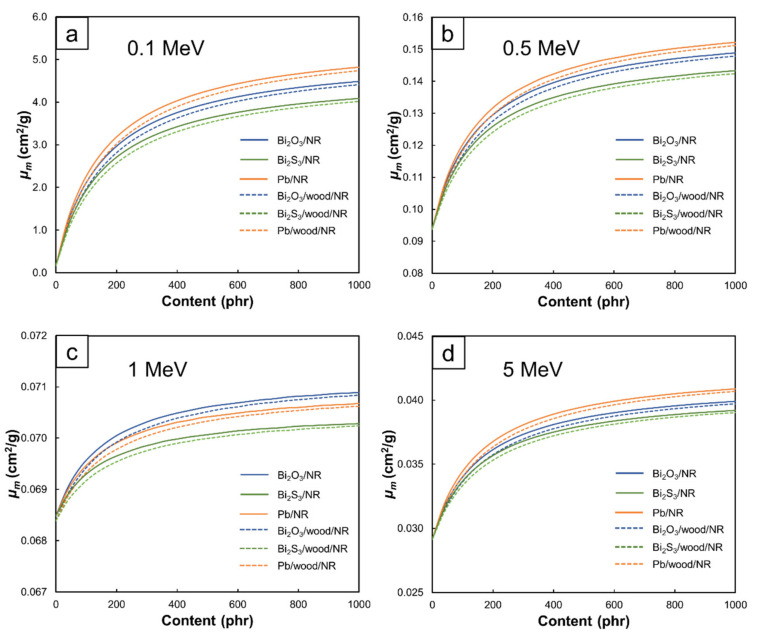
*µ_m_* values of Bi_2_O_3_/NR, Bi_2_O_3_/wood/NR, Bi_2_S_3_/NR, Bi_2_S_3_/wood/NR, Pb/NR, and Pb/wood/NR composites with filler contents varied from 0–1000 phr, determined at photon energies of (**a**) 0.1 MeV, (**b**) 0.5 MeV, (**c**) 1 MeV, and (**d**) 5 MeV using XCOM.

The *µ_m_* values at photon energies of 0.001–5 MeV for Bi_2_O_3_/NR, Bi_2_S_3_/NR, Pb/NR, Bi_2_O_3_/wood/NR, Bi_2_S_3_/wood/NR, and Pb/wood/NR composites with varying filler contents of 0, 400, and 800 phr are shown in Figure 1, Figure 2, Figure 3 and Figure 4. The results indicated that the composites with the addition of Bi_2_O_3_, Bi_2_S_3_, or Pb had noticeably higher *µ_m_* values than the pristine NR and wood/NR composites at all investigated photon energies (Figure 1, Figure 2 and Figure 3), with the *µ_m_* values increased with increasing filler contents (Figure 4). This could have resulted from the added Bi_2_O_3_ (Bi_2_S_3_), which contained Bi atoms, and the Pb particles greatly enhanced the interaction probabilities between the incident photons and the composites through photoelectric absorption, Compton scattering, and pair production (pair production could occur only at photon energies greater than 1.022 MeV) due to their high *Z* and *ρ* values, with the relationships between the cross sections for each mechanism and its photon/material characteristics being shown as Equations (6)–(8):

(6) $$\sigma_{pe}\propto \frac{Z^{n}}{{\left(h\nu \right)}^{3}}$$

(7) $$\sigma_{comp}\propto \frac{1}{n_{e}}$$

(8) $$\sigma_{pp}\propto Z^{2}$$

where σ*_pe_* is the photoelectric cross section, σ*_comp_* is the Compton scattering cross section, σ*_pp_* is the pair production cross section, Z is the atomic number of the element, *h* is Planck’s constant, *ν* is the frequency of the photon, and *n_e_* is the electron density, which is related to density and *Z* of the material [27].

**Figure 5:**
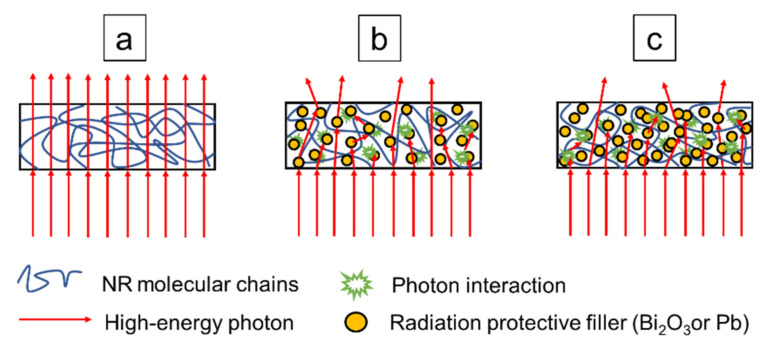
Scheme showing interactions of incident photons and NR composites for (**a**) a pristine NR, (**b**) and (**c**) NR composites containing radiation protective fillers (filler contents of (**c**) greater than (**b**)).

The effects of Bi_2_O_3_ and Pb and their contents on enhancing high-energy photon attenuation are graphically illustrated in Figure 5.

**Figure 6:**
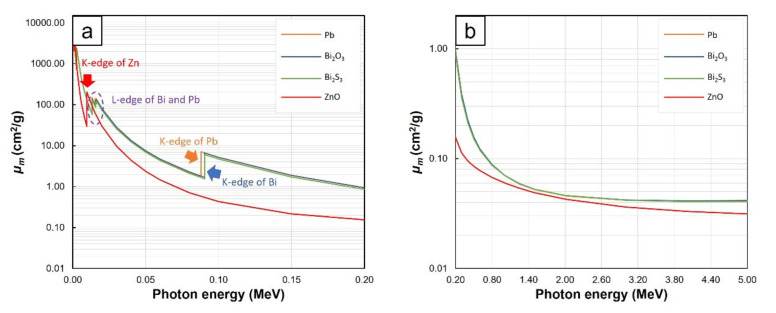
*µ_m_* values of Pb, Bi_2_O_3_, Bi_2_S_3_, and ZnO, showing K-edge and L-edge behavior of Pb, Bi, and Zn at photon energies of (**a**) 0.001–0.2 MeV and (**b**) 0.2–5 MeV.

Furthermore, the results in Figure 1, Figure 2 and Figure 3 revealed that *µ_m_* values tended to decrease with increasing photon energies and the effects of fillers on *µ_m_* were more pronounced at lower energies. These behaviors were observed because higher-energy photons were less likely to interact with materials they encountered than lower-energy photons due to the inverse relationship between linear energy transfer (*LET*) and photon energies [28], leading to less energy loss or absorption and consequently, less photon attenuation and *µ_m_* values. Interestingly, there were sharp increases in the values of *µ_m_* in NR composites containing Bi_2_O_3_, Bi_2_S_3_, and Pb at photon energies of:

- 13–16. keV and 90.5 keV in Bi_2_O_3_/NR, Bi_2_S_3_/NR, Bi_2_O_3_/wood/NR, and Bi_2_S_3_/wood/NR composites (Figure 1a,c and Figure 2a,c) due to the L-edge and K-edge of Bi atoms in Bi_2_O_3_ and Bi_2_S_3_, respectively (Figure 6)
- 13–16. keV and 88.0 keV in Pb/NR and Pb/wood/NR composites (Figure 3a,c) due to the L-edge and K-edge of Pb atoms, respectively (Figure 6)

And in all composites at the photon energy of 9.6 keV (Figure 1a,c, Figure 2a,c, and Figure 3a,c) due to the K-edge of Zn atoms in ZnO (Figure 6). At these uncharacteristically high values of *µ_m_*, the photon energies were just above the binding energy of the electron shells inside the atoms (the binding energy of the electrons at K-shell and L-shell were called K-edge and L-edge, respectively), resulting in substantially enhanced probabilities of photon interaction through photoelectric absorption at these particular energies [29]. It is noteworthy that values of *µ_m_* in pristine NR were higher than those in Bi_2_O_3_/NR, Bi_2_S_3_, and Pb/NR at the photon energies of 1–2 MeV. These behaviors were due to the rapid drops in photoelectric absorption and Compton scattering probabilities of Bi_2_O_3_, Bi_2_S_3_, and Pb particles at these mid-ranged photon energies. These behaviors were in contrast with the decreases in interaction probabilities of C and H that were much less pronounced (*µ_m_* of NR was actually higher than Bi_2_O_3_, Bi_2_S_3_, and Pb at these energy range). However, pair- production probability of Bi_2_O_3_, Bi_2_S_3_, and Pb sharply increases at photon energies greater than 2 MeV, resulting in much enhanced interaction probabilities of composites containing Bi_2_O_3_, Bi_2_S_3_, and Pb that consequently led to higher *µ_m_* in Bi_2_O_3_/NR, Bi_2_S_3_/NR, and Pb/NR composites (pair production cross sections (probabilities) of light elements were relatively smaller than those of heavy elements).

Figure 4 also revealed that the Pb/NR composites generally had higher *µ_m_* values than the Bi_2_O_3_/NR and Bi_2_S_3_/NR composites for the same filler content and photon energy (Figure 4a,b,d). This was due to the higher *µ_m_* of Pb compared to Bi (Figure 6) and the dilution effect of O and S in Bi_2_O_3_ and Bi_2_S_3_, respectively that further reduced influences of Bi. However, the *µ_m_* values of the Bi_2_O_3_/NR composites shown in Figure 4c were slightly higher than those of the Pb/NR composites at a photon energy of 1 MeV because, for this particular range of energies, Bi_2_O_3_ had slightly higher *µ_m_* values than Pb (0.0713 and 0.0710 cm^2^/g, respectively), resulting in higher attenuation abilities and consequently, higher *µ_m_* values in the Bi_2_O_3_/NR composites. In addition, comparing *µ_m_* values of NR and wood/NR composites with the addition of Bi_2_O_3_ and Bi_2_S_3_ at the same filler content and photon energy, Figure 4 revealed that those with Bi_2_O_3_ exhibited slightly higher µ_m_ than those with Bi_2_S_3_. This could be due to higher mass fraction of Bi in a molecule of Bi_2_O_3_ (0.89) than Bi_2_S_3_ (0.81), which resulted in higher numbers of Bi atoms in Bi_2_O_3_ than Bi_2_S_3_ at the same filler content.

To validate the simulated results from XCOM in this work, previously reported experimental values were used to compare their agreements. The comparisons of the two values for the NR composites containing different filler types/contents at various photon energies are shown in Table 2. The percentage difference between the two values (Difference (%)) was calculated based on Equation (9):

(9) $$\mathrm{Difference}\;\left(\%\right)=\;\frac{\left|\mu_{m,XCOM}-\mu_{m,report}\right|}{\mu_{m,XCOM}}\times 100\%$$

where Difference (%) is the percentage of difference between simulated result from XCOM and the previously reported experimental value, *µ_m,XCOM_* is the µ_m_ value from the simulated result, *µ_m,report_* is the µ_m_ value from the previously reported experimental value.

**Table 2:** Comparative *µ_m_* values from simulated data using XCOM and previously reported experimental results of NR composites containing different filler types/contents at various photon energies.

Filler Type	Content (phr)	Photon Energy (MeV)	*µ_m_* (cm^2^/g)			Reference
			XCOM	Literature Value	Difference (%)	
Pb	0	0.122	0.162	0.159	1.9	[30]
	40		0.943	0.813	13.8	
	80		1.459	1.198	17.9	
Pb	0	0.356	0.108	0.106	1.9	[30]
	40		0.149	0.142	4.7	
	80		0.176	0.166	5.7	
Pb	0	0.662	0.084	0.086	2.4	[30]
	40		0.090	0.089	1.1	
	80		0.094	0.091	3.2	
Pb	100	0.662	0.095	0.085	10.5	[22]
	300		0.103	0.094	8.7	
	500		0.106	0.096	9.4	
	1000		0.110	0.092	16.4	
Pb	0	1.173	0.063	0.064	1.6	[30]
	40		0.063	0.063	0.0	
	80		0.063	0.063	0.0	
Pb	100	1.250	0.060	0.051	15.0	[22]
	300		0.060	0.052	13.3	
	500		0.059	0.056	5.1	
	1000		0.059	0.051	13.6	
Pb	0	1.332	0.059	0.059	0.0	[30]
	40		0.059	0.058	1.7	
	80		0.059	0.058	1.7	
Bi_2_O_3_	0	0.223	0.129	0.130	0.2	[31]
	20		0.206	0.225	8.8	
	40		0.255	0.289	13.4	
	80		0.342	0.382	11.8	
	150		0.463	0.469	1.4	
Bi_2_O_3_	0	0.253	0.124	0.123	0.8	[31]
	20		0.183	0.190	3.8	
	40		0.220	0.237	7.7	
	80		0.286	0.304	6.3	
	150		0.379	0.371	2.1	
Bi_2_O_3_	0	0.341	0.110	0.112	1.8	[31]
	20		0.134	0.138	3.0	
	40		0.148	0.163	10.1	
	80		0.175	0.188	7.4	
	150		0.212	0.216	1.9	
Bi_2_O_3_	0	0.482	0.095	0.096	1.1	[31]
	20		0.104	0.106	1.9	
	40		0.109	0.116	6.4	
	80		0.119	0.124	4.2	
	150		0.132	0.137	3.8	
Bi_2_O_3_	0	0.662	0.083	0.083	0.0	[31]
	20		0.087	0.088	1.2	
	40		0.089	0.091	2.3	
	80		0.092	0.095	3.3	
	150		0.098	0.097	1.0	

As shown in Table 2, the range for Difference (%) was 0.0–17.9% with the average value being 5.3%. This comparison clearly showed that the simulated results from XCOM were in acceptable agreement with the experimental data; hence, they could be reliably used in later investigations of *µ*, *HVL*, and the recommended filler contents in this work. It should be pointed out that the differences in the two values could have been due to several factors, including experimental setup and equipment that caused an imperfectly monoenergetic and narrow photon beam, agglomerations of fillers, voids at the interfacial surfaces of the NR matrix and filler particles, and build-up factors that lowered the experimental *µ_m_* values to be generally less than the theoretical ones [13,32].

### 3.2. Linear Attenuation Coefficients (µ) and Half Value Layer (HVL)

To determine the *µ* and *HVL* values of the Bi_2_O_3_/NR, Bi_2_S_3_/NR, Pb/NR, Bi_2_O_3_/wood/NR, Bi_2_S_3_/wood/NR, and Pb/wood/NR composites, the densities for each filler content must be estimated. The results of the density calculations using Equation 3 are shown in Table 3, which indicates that densities generally increased with increasing filler contents and the NR composites containing Pb had slightly higher densities than those with Bi_2_O_3_ and Bi_2_S_3_ due to the higher density of Pb (11.35 g/cm^3^) compared to Bi_2_O_3_ (8.90 g/cm^3^) and Bi_2_S_3_ (6.78 g/cm^3^). Furthermore, Table 3 shows that the NR composites with the addition of wood particles had lower densities than those without wood particles (for the same filler content), mainly due to the much lower density of the wood particles (0.52 g/cm^3^) compared to NR (0.92 g/cm^3^), Bi_2_O_3_, Bi_2_S_3_, and Pb. It should be noted that the densities of actual NR and wood/NR composites might differ from the theoretical densities shown in Table 2 due to possible aggregation of fillers, poor interfacial compatibility between fillers and NR matrix, and incomplete vulcanization of NR from the obstruction of fillers during the curing process.

**Figure 7:**
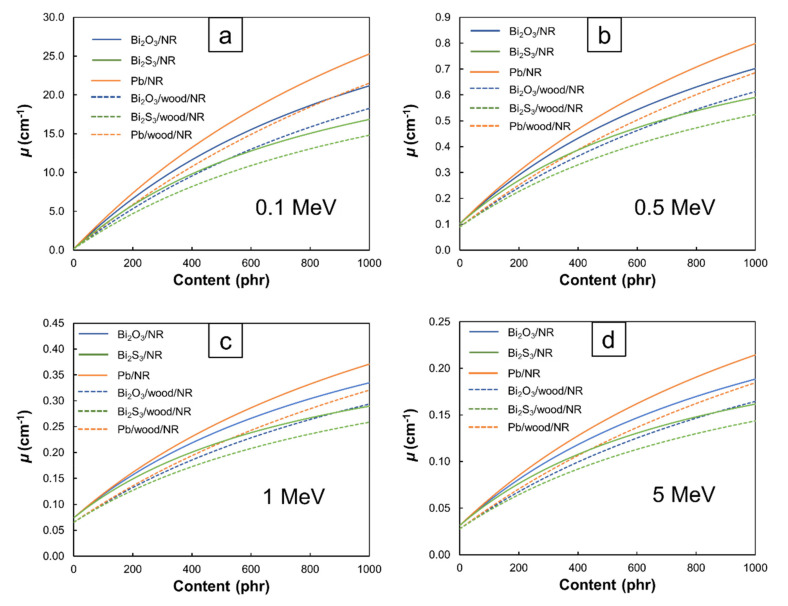
*µ* values of Bi_2_O_3_/NR, Bi_2_S_3_/NR, Pb/NR, Bi_2_O_3_/wood/NR, Bi_2_S_3_/wood/NR, and Pb/wood/NR composites at photon energies of (**a**) 0.1 MeV, (**b**) 0.5 MeV, (**c**) 1 MeV, and (**d**) 5 MeV.

**Figure 8:**
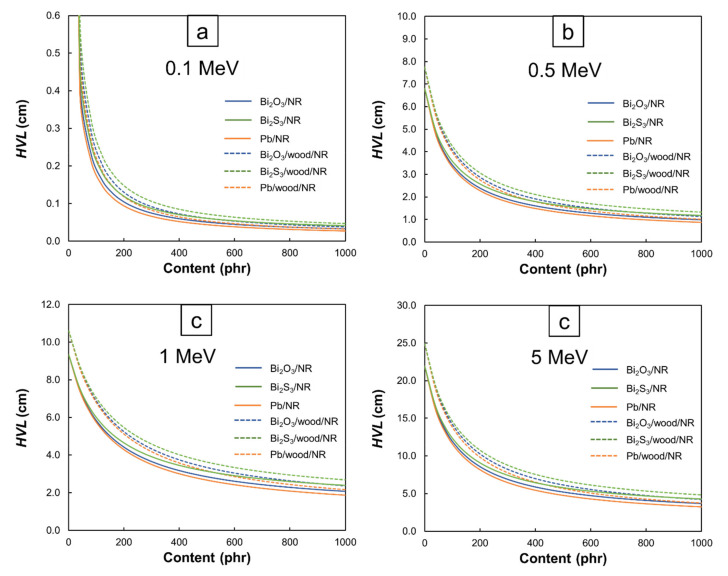
*HVL* values of Bi_2_O_3_/NR, Bi_2_S_3_/NR, Pb/NR, Bi_2_O_3_/wood/NR, Bi_2_S_3_/wood/NR, and Pb/wood/NR composites at photon energies of (**a**) 0.1 MeV, (**b**) 0.5 MeV, (**c**) 1 MeV, and (**d**) 5 MeV.

Figure 7 and Figure 8 illustrate the values of *µ* and *HVL* of the Bi_2_O_3_/NR, Bi_2_S_3_/NR, Pb/NR, Bi_2_O_3_/wood/NR, Bi_2_S_3_/wood/NR, and Pb/wood/NR composites with varying filler contents and photon energies. The results indicated that *µ* tended to increase but *HVL* tended to decrease with increasing filler contents. These results agreed with the trends found in *µ_m_* (Figure 1, Figure 2, Figure 3 and Figure 4) due to the capability of Bi and Pb atoms to enhance the interaction probabilities between incident photons and the composites, leading to higher photon attenuation per unit length (*µ*), and consequently, less material thickness being required to attenuate 50% of the incident photon intensity (*HVL*). Additionally, Figure 7 shows that the Pb/NR composites had relatively higher *µ* values than the Bi_2_O_3_/NR composites for the same filler content and photon energy, mainly due to the former having greater density and *µ_m_* than the latter (Equation (1)). Similarly, the NR composites with the addition of wood particles had lower *µ* but higher *HVL* values than those without wood particles. This behavior was observed due to the low density of the wood particles (Table 1) that generally decreased the density of the NR composites (Table 3) as well as the relatively low *µ_m_* of wood particles compared to Bi_2_O_3_, Bi_2_S_3_, and Pb. Nonetheless, although the shielding properties of the NR composites containing Bi_2_O_3_ (Bi_2_S_3_) were reduced by as much as 16% (33%) at a photon energy of 0.1 MeV and by 5% (24%) at a photon energy of 5 MeV (determined at 1000 phr, which was the highest content investigated in this work), the potential of using bismuth compounds, especially Bi_2_O_3_, as an alternative but safer protective filler was promising. In addition, the *µ* (*HVL*) values at lower energies were generally higher (lower) than those at higher energies and the effects of additional filler contents on enhancing shielding abilities were more pronounced at lower energies than at higher energies (determined for the same type of composites). This could have been due to the higher-energy photons being less likely to interact with the materials they encountered (Equations (6)–(8)), leading to less attenuation and therefore, smaller (larger) *µ* (*HVL*) values.

### 3.3. Recommended Contents of Bi_2_O_3_, Bi_2_S_3_, and Pb in NR and Wood/NR Composites

**Figure 9:**
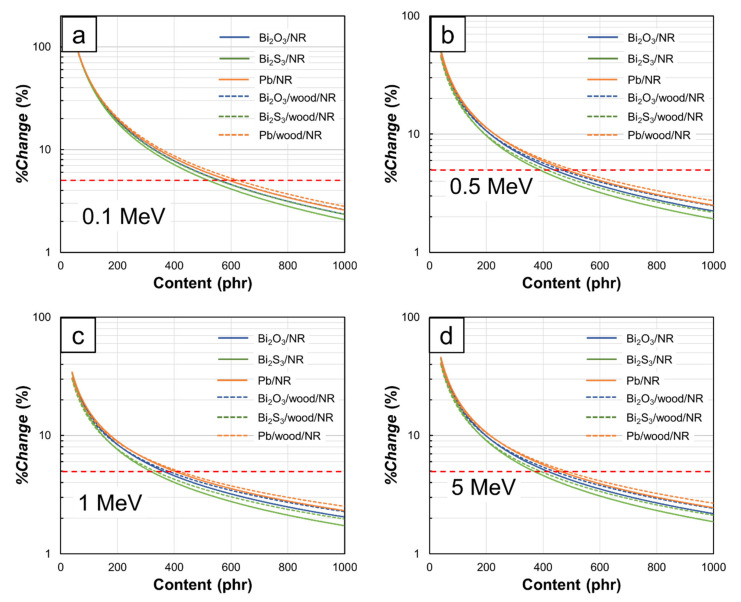
*%change* in *µ* values at photon energies of (**a**) 0.1 MeV, (**b**) 0.5 MeV, (**c**) 1 MeV, and (**d**) 5 MeV after addition of 40-phr Bi_2_O_3_, Bi_2_S_3_, or Pb into Bi_2_O_3_/NR, Bi_2_S_3_/NR, Pb/NR, Bi_2_O_3_/wood/NR, Bi_2_S_3_/wood/NR, and Pb/wood/NR composites. Horizontal dotted lines represent the threshold set in this work to determine recommended filler contents (%*change* < 5%).

To determine the recommended filler contents for the Bi_2_O_3_/NR, Bi_2_S_3_/NR, Pb/NR, Bi_2_O_3_/wood/NR, Bi_2_S_3_/wood/NR, and Pb/wood/NR composites at different photon energies, the values of *%change* in *µ* were calculated using Equation 4 and the results are shown in Figure 9. The results indicated that the recommended contents for Bi_2_O_3_, Bi_2_S_3_, and Pb were in the ranges 358–594 phr, 316–557 phr, and 398–624 phr, respectively, depending on photon energies and the present of wood particles. Furthermore, it is noteworthy that values of *%change* were initially much higher at low filler contents due to the more pronounced effects of Bi and Pb in enhancing shielding properties from their relatively higher cross sections for photoelectric absorption and Compton scattering than C, H, and O in the pristine NR and wood/NR composites. This effect led to a significant enhancement in photon attenuation of the NR composites at low photon energies. However, as more fillers were added to the composites, the effects of fillers on *%change* were less pronounced and essentially became less than 5% (a threshold set in this work for the determination of recommended filler contents).

To obtain the recommended filler contents for all photon energies, fitted curves of *%change* with the form shown in Equation (5) were developed and mathematical constants (A and B) were obtained using a trendline function available in Microsoft Excel. The values of A and B as well as recommended filler contents for photon energies of 0.1 MeV, 0.5 MeV, 1 MeV, and 5 MeV (based on the threshold of 5%) determined from the fitted curves are shown in Table 4, which indicated that lower photon energies (0.1 MeV and 0.5 MeV) required higher filler contents to achieve less than 5% of *%change* than those at higher photon energies (1 MeV and 5 MeV). This behavior was observed because Bi_2_O_3,_ Bi_2_S_3_, and Pb could better interact with photons at lower energies, leading to greater shielding enhancement after adding more fillers to the composites.

Table 4 also shows the effects of potentially replacing Pb with Bi_2_O_3_ or Bi_2_S_3_ by considering the percentage difference in *µ* values of Pb and Bi_2_O_3_ (Bi_2_S_3_) at the determined recommended contents. The results indicated that the differences were in the range 8.3–16.3% for those with Bi_2_O_3_ and 19.6–28.4% for those with Bi_2_S_3_, of which NR and wood/NR composites containing Pb had higher *µ* values than those with Bi_2_O_3_ and Bi_2_S_3_. Nonetheless, as a considerably safer chemical than Pb, bismuth compounds, especially Bi_2_O_3_, showed their potential to replace the former as effective radiation-protective fillers.

Interestingly, Table 4 reveals that the recommended filler contents for 1 MeV photons were the lowest among all energies. This could be explained using Figure 4c, which indicates that the *µ_m_* values for all filler contents were not greatly different (0.068–0.071 cm^2^/g), resulting in lower filler contents being required to obtain <5% of *%change*. On the other hand, the results pointed out that higher filler contents were required to achieve the same *%change* at the 5 MeV photon energy, mostly due to the initiation of pair production, which again enhanced the effects of added Bi_2_O_3_, Bi_2_S_3_, and Pb in photon attenuation, as seen by larger changes in *µ_m_* when the filler contents increased from 0 to 1000 phr (*µ_m_* increased from 0.029 to 0.040 cm^2^/g). It should be noted that the recommended filler contents given in this work were not intended for the highest µ of the composites but rather the least amount of filler that produced a *%change* of an additional 40 phr filler to be less than 5% in order to minimize the effects of having too much filler in the NR matrix and its impact on lowering mechanical properties [13,20,24,31].

## 4. Conclusions

This work determined the theoretical high-energy photon shielding properties consisting of *µ_m_*, *µ*, and *HVL* as well as the recommended filler contents for Bi_2_O_3_/NR, Bi_2_S_3_/NR, Pb/NR, Bi_2_O_3_/wood/NR, Bi_2_S_3_/wood/NR, and Pb/wood/NR composites with the filler contents in the range 0–1000 phr and photon energies in the range 0.001–5 MeV for the development of X-ray and gamma-shielding equipment with flexibility and enhanced dimensional stability. The XCOM simulation software was used in this work and was provided by NIST, USA. The results showed that values of *µ_m_* and *µ* (*HVL*) increased (decreased) with increasing filler contents but decreased (increased) with increasing photon energies. Furthermore, the addition of wood particles in the wood/NR composites did not greatly affect the overall shielding properties compared with the NR composites, implying the possibility of applying wood fillers as a dimensional reinforcer for composites with high filler contents. In addition, Bi_2_O_3_ and Bi_2_S_3_ also showed great potential to replace Pb as a high-energy-photon protective filler due to its relatively high attenuation efficiency and being considerably less hazardous than Pb. Lastly, the determination of the recommended filler contents based on calculation of the %*change* in *µ* indicated that the optimum Pb, Bi_2_O_3_, or Bi_2_S_3_ contents at photon energies of 0.1, 0.5, 1, and 5 MeV were in the range 316–624 phr, depending on the filler type and the intended photon energy.

## Figures and Tables

**Table 1:** Name, chemical formula, content, and density of chemicals used for the simulation in this work [14,20].

Chemical	Chemical Formula	Content (phr *)	Density (g/cm^3^)
Natural rubber (NR)	C_5_H_8_	100	0.92
Stearic acid	C_18_H_36_O_2_	3	0.94
Zinc oxide	ZnO	5	5.6
Mercaptobenzothiazole (MBT)	C_7_H_5_NS_2_	2	1.46
Diphenylguanidine (DPG)	C_13_H_13_N_3_	3	1.19
Carbon black	C	40	1.95
Sulfur	S	4	2.1
Wood particles (cellulose)	C_6_H_10_O_5_	0 or 20	0.52
Bismuth oxide, bismuth sulfide, or lead	Bi_2_O_3_, Bi_2_S_3_ or Pb	0–1000	8.90, 6.78 or 11.35

* phr: parts per hundred parts of rubber by weight.

**Table 3:** Densities of Bi_2_O_3_/NR, Bi_2_S_3_/NR, Pb/NR, Bi_2_O_3_/wood/NR, Bi_2_S_3_/wood/NR, and Pb/wood/NR composites with filler contents varying from 0 to 1000 phr (in 40-phr increments), calculated using Equation (3).

Content (phr)	Density (g/cm^3^)					
	Bi_2_O_3_/NR	Bi_2_S_3_/NR	Pb/NR	Bi_2_O_3_/Wood/NR	Bi_2_S_3_/Wood/NR	Pb/Wood/NR
0	1.09	1.08	1.08	0.96	0.95	0.95
40	1.36	1.33	1.36	1.17	1.15	1.17
80	1.61	1.56	1.61	1.37	1.34	1.37
120	1.84	1.77	1.86	1.56	1.51	1.57
160	2.05	1.96	2.09	1.74	1.67	1.76
200	2.26	2.14	2.32	1.91	1.83	1.94
240	2.45	2.31	2.53	2.07	1.97	2.12
280	2.63	2.46	2.73	2.22	2.11	2.29
320	2.80	2.61	2.92	2.37	2.23	2.45
360	2.97	2.74	3.11	2.51	2.36	2.61
400	3.12	2.87	3.28	2.65	2.47	2.76
440	3.27	2.99	3.45	2.78	2.58	2.91
480	3.41	3.10	3.62	2.90	2.68	3.05
520	3.54	3.21	3.77	3.02	2.78	3.19
560	3.67	3.30	3.92	3.14	2.88	3.32
600	3.79	3.40	4.06	3.25	2.97	3.45
640	3.90	3.49	4.20	3.36	3.05	3.57
680	4.01	3.57	4.34	3.46	3.14	3.69
720	4.12	3.65	4.46	3.56	3.20	3.81
760	4.22	3.73	4.59	3.65	3.29	3.92
800	4.31	3.80	4.71	3.74	3.36	4.03
840	4.41	3.87	4.82	3.83	3.43	4.14
880	4.50	3.94	4.93	3.92	3.50	4.24
920	4.58	4.00	5.04	4.00	3.56	4.34
960	4.66	4.06	5.15	4.08	3.62	4.44
1000	4.74	4.12	5.25	4.16	3.68	4.54

**Table 4:** Mathematical constants from Figure 9, recommended filler contents at photon energy of 0.1–5 MeV (*%change* < 5%), and percentage of difference between NR (wood/NR) composites containing Bi_2_O_3_, Bi_2_S_3_, and Pb.

Composite	Photon Energy (MeV)	Coefficient		Recommended Content (phr)	*µ* at Recommended Content (cm^−1^)	Percentage Difference from Composites with Pb (%) *
		A	B			
Pb/NR	0.1	1.652 × 10^4^	−1.269	593	17.8	n/a **
	0.5	1.664 × 10^3^	−0.941	478	0.52	n/a
	1	760.3	−0.839	398	0.23	n/a
	5	1.312 × 10^3^	−0.909	458	0.14	n/a
Bi_2_O_3_/NR	0.1	1.894 × 10^4^	−1.303	557	14.9	16.3
	0.5	1.839 × 10^3^	−0.972	436	0.46	11.5
	1	869.4	−0.877	358	0.21	8.7
	5	1.439 × 10^3^	−0.940	413	0.12	14.3
Bi_2_S_3_/NR	0.1	2.271 × 10^4^	−1.346	521	12.8	28.4
	0.5	2.011 × 10^3^	−1.007	386	0.39	24.6
	1	985.0	−0.918	316	0.18	20.1
	5	1.623 × 10^3^	−0.980	365	0.11	24.8
Pb/wood/NR	0.1	1.389 × 10^4^	−1.232	624	15.3	n/a
	0.5	1.199 × 10^3^	−0.881	502	0.44	n/a
	1	535.9	−0.776	413	0.20	n/a
	5	0.937 × 10^3^	−0.848	476	0.12	n/a
Bi_2_O_3_/wood/NR	0.1	1.553 × 10^4^	−1.259	594	12.8	16.3
	0.5	1.303 × 10^3^	−0.907	461	0.40	9.1
	1	600.5	−0.808	375	0.18	10.0
	5	1.008 × 10^3^	−0.874	434	0.11	8.3
Bi_2_S_3_/wood/NR	0.1	1.812 × 10^4^	−1.297	557	11.2	26.6
	0.5	1.402 × 10^3^	−0.937	410	0.35	21.5
	1	670.5	−0.844	332	0.16	19.6
	5	1.121 × 10^3^	−0.908	388	0.09	22.1

* Determined with respect to Pb/NR or Pb/wood/NR composites at the same photon energy. ** n/a: not applicable.

## Supplementary Materials

The following are available online at https://www.mdpi.com/2073-4360/13/6/869/s1, Table S1: Mass attenuation coefficients (*µ_m_*) of NR composites, Table S2: Mass attenuation coefficients (*µ_m_*) of 400-phr-Bi_2_O_3_/NR composites, Table S3: Mass attenuation coefficients (*µ_m_*) of 800-phr-Bi_2_O_3_/NR composites, Table S4: Mass attenuation coefficients (*µ_m_*) of 400-phr-Pb/NR composites, Table S5: Mass attenuation coefficients (*µ_m_*) of 800-phr-Pb/NR composites, Table S6: Mass attenuation coefficients (*µ_m_*) of 400-phr-Bi_2_S_3_/NR composites, Table S7: Mass attenuation coefficients (*µ_m_*) of 800-phr-Bi_2_S_3_/NR composites, Table S8: Mass attenuation coefficients (*µ_m_*) of wood/NR composites, Table S9: Mass attenuation coefficients (*µ_m_*) of 400-phr-Bi_2_O_3_/wood/NR composites, Table S10: Mass attenuation coefficients (*µ_m_*) of 800-phr-Bi_2_O_3_/wood/NR composites, Table S11: Mass attenuation coefficients (*µ_m_*) of 400-phr-Pb/wood/NR composites, Table S12: Mass attenuation coefficients (*µ_m_*) of 800-phr-Pb/wood/NR composites, Table S13: Mass attenuation coefficients (*µ_m_*) of 400-phr-Bi_2_S_3_/wood/NR composites, Table S14: Mass attenuation coefficients (*µ_m_*) of 800-phr-Bi_2_S_3_/wood/NR composites.
