# Supplementary material for: Theoretical Determination of High-Energy Photon Attenuation and Recommended Protective Filler Contents for Flexible and Enhanced Dimensionally Stable Wood/NR and NR Composites
Source: Polymers (Basel). 2021 Mar 11;13(6):869. doi: 10.3390/polym13060869 (PMC7998293; doi:10.3390/polym13060869)
Supplement: Supplementary file 1 [file polymers-13-00869-s001.pdf]

Supporting Information

Theoretical determination of high-energy photon attenuation and recommended protective-filler contents for flexible and enhanced dimensionally stable wood/NR and NR composites

Worawat Poltabtim [1], Donruedee Toyen [2] and Kiadtisak Saenboonruang [1,3]*

[1] Department of Applied Radiation and Isotopes, Faculty of Science, Kasetsart University, Bangkok, Thailand 10900; wp.worawat@gmail.com (W.P.); kiadtisak.s@ku.th (K.S.)
[2] Scientific Equipment and Research Division, Kasetsart University Research and Development Institute (KURDI), Kasetsart University, Bangkok, Thailand 10900; rdiddt@ku.ac.th (D.T.)
[3] Specialized Center of Rubber and Polymer Materials in Agriculture and Industry (RPM), Faculty of Science, Kasetsart University, Bangkok, Thailand 10900; kiadtisak.s@ku.th (K.S.)
* Correspondence: kiadtisak.s@ku.th; Tel.: +662-562-5555 ext. 1217

Table S1. Mass attenuation coefficients (μ_m) of NR composites.

Photon energy (MeV)	μ_m (cm^2/g)
1.00E-03	2.05E+03
1.01E-03	2.00E+03
1.02E-03	1.94E+03
1.02E-03	2.00E+03
1.03E-03	1.97E+03
1.04E-03	1.96E+03
1.04E-03	2.00E+03
1.12E-03	1.68E+03
1.19E-03	1.41E+03
1.19E-03	1.44E+03
1.50E-03	7.64E+02
2.00E-03	3.38E+02
2.47E-03	1.84E+02
2.47E-03	2.40E+02
3.00E-03	1.41E+02
4.00E-03	6.24E+01
5.00E-03	3.29E+01
6.00E-03	1.94E+01
8.00E-03	8.43E+00
9.66E-03	4.92E+00
9.66E-03	1.05E+01
1.00E-02	9.60E+00
1.50E-02	3.28E+00
2.00E-02	1.57E+00
3.00E-02	6.24E-01
4.00E-02	3.75E-01
5.00E-02	2.80E-01
6.00E-02	2.35E-01
8.00E-02	1.93E-01
1.00E-01	1.73E-01
1.50E-01	1.48E-01
2.00E-01	1.34E-01
3.00E-01	1.15E-01
4.00E-01	1.03E-01
5.00E-01	9.39E-02
6.00E-01	8.68E-02
8.00E-01	7.62E-02
1.00E+00	6.85E-02
1.02E+00	6.78E-02
1.25E+00	6.13E-02
1.50E+00	5.58E-02
2.00E+00	4.78E-02
2.04E+00	4.73E-02
3.00E+00	3.84E-02
4.00E+00	3.28E-02

5.00E+00	2.92E-02

Table S2. Mass attenuation coefficients (μ_m) of 400-phr-Bi_2O_3/NR composites.

Photon energy (MeV)	μ_m (cm^2/g)
1.00E-03	4.42E+03
1.01E-03	4.33E+03
1.02E-03	4.24E+03
1.02E-03	4.26E+03
1.03E-03	4.17E+03
1.04E-03	4.08E+03
1.04E-03	4.10E+03
1.12E-03	3.56E+03
1.19E-03	3.09E+03
1.19E-03	3.10E+03
1.50E-03	1.92E+03
2.00E-03	1.02E+03
2.47E-03	6.26E+02
2.47E-03	6.42E+02
2.53E-03	6.11E+02
2.58E-03	5.83E+02
2.58E-03	1.23E+03
2.63E-03	1.25E+03
2.69E-03	1.27E+03
2.69E-03	1.74E+03
3.00E-03	1.38E+03
3.18E-03	1.19E+03
3.18E-03	1.37E+03
3.43E-03	1.13E+03
3.70E-03	9.42E+02
3.70E-03	9.96E+02
3.85E-03	9.06E+02
4.00E-03	8.25E+02
4.00E-03	8.60E+02
4.00E-03	8.59E+02
5.00E-03	5.01E+02
6.00E-03	3.20E+02
8.00E-03	1.56E+02
9.66E-03	9.74E+01
9.66E-03	9.90E+01
1.00E-02	9.07E+01
1.34E-02	4.32E+01
1.34E-02	1.02E+02
1.50E-02	7.57E+01
1.57E-02	6.71E+01
1.57E-02	9.21E+01
1.61E-02	8.77E+01
1.64E-02	8.34E+01

1.64E-02	9.60E+01
2.00E-02	5.82E+01
3.00E-02	2.05E+01
4.00E-02	9.75E+00
5.00E-02	5.49E+00
6.00E-02	3.45E+00
8.00E-02	1.69E+00
9.05E-02	1.26E+00
9.05E-02	4.82E+00
1.00E-01	3.76E+00
1.50E-01	1.39E+00
2.00E-01	7.12E-01
3.00E-01	3.09E-01
4.00E-01	1.90E-01
5.00E-01	1.40E-01
6.00E-01	1.13E-01
8.00E-01	8.49E-02
1.00E+00	7.05E-02
1.02E+00	6.93E-02
1.25E+00	5.98E-02
1.50E+00	5.36E-02
2.00E+00	4.68E-02
2.04E+00	4.64E-02
3.00E+00	4.10E-02
4.00E+00	3.89E-02
5.00E+00	3.81E-02

Table S3. Mass attenuation coefficients (μ_m) of 800-phr-Bi_2O_3/NR composites.

Photon energy (MeV)	μ_m (cm^2/g)
1.00E-03	4.81E+03
1.01E-03	4.72E+03
1.02E-03	4.62E+03
1.02E-03	4.63E+03
1.03E-03	4.53E+03
1.04E-03	4.43E+03
1.04E-03	4.44E+03
1.12E-03	3.87E+03
1.19E-03	3.37E+03
1.19E-03	3.37E+03
1.50E-03	2.11E+03
2.00E-03	1.13E+03
2.47E-03	6.98E+02
2.47E-03	7.08E+02
2.53E-03	6.75E+02
2.58E-03	6.43E+02
2.58E-03	1.40E+03
2.63E-03	1.42E+03

2.69E-03	1.45E+03
2.69E-03	1.99E+03
3.00E-03	1.58E+03
3.18E-03	1.37E+03
3.18E-03	1.57E+03
3.43E-03	1.30E+03
3.70E-03	1.08E+03
3.70E-03	1.15E+03
3.85E-03	1.04E+03
4.00E-03	9.50E+02
4.00E-03	9.91E+02
4.00E-03	9.90E+02
5.00E-03	5.78E+02
6.00E-03	3.70E+02
8.00E-03	1.81E+02
9.66E-03	1.13E+02
9.66E-03	1.14E+02
1.00E-02	1.04E+02
1.34E-02	4.96E+01
1.34E-02	1.18E+02
1.50E-02	8.76E+01
1.57E-02	7.76E+01
1.57E-02	1.07E+02
1.61E-02	1.02E+02
1.64E-02	9.67E+01
1.64E-02	1.11E+02
2.00E-02	6.74E+01
3.00E-02	2.38E+01
4.00E-02	1.13E+01
5.00E-02	6.35E+00
6.00E-02	3.98E+00
8.00E-02	1.94E+00
9.05E-02	1.44E+00
9.05E-02	5.58E+00
1.00E-01	4.34E+00
1.50E-01	1.60E+00
2.00E-01	8.07E-01
3.00E-01	3.40E-01
4.00E-01	2.04E-01
5.00E-01	1.47E-01
6.00E-01	1.17E-01
8.00E-01	8.64E-02
1.00E+00	7.08E-02
1.02E+00	6.96E-02
1.25E+00	5.96E-02
1.50E+00	5.32E-02
2.00E+00	4.66E-02
2.04E+00	4.62E-02

3.00E+00	4.15E-02
4.00E+00	3.99E-02
5.00E+00	3.96E-02

Table S4. Mass attenuation coefficients (μ_m) of 400-phr-Pb/NR composites.

Photon energy (MeV)	μ_m (cm^2/g)
1.00E-03	4.32E+03
1.01E-03	4.23E+03
1.02E-03	4.15E+03
1.02E-03	4.16E+03
1.03E-03	4.08E+03
1.04E-03	4.00E+03
1.04E-03	4.01E+03
1.12E-03	3.49E+03
1.19E-03	3.04E+03
1.19E-03	3.05E+03
1.50E-03	1.91E+03
2.00E-03	1.02E+03
2.47E-03	6.33E+02
2.47E-03	6.49E+02
2.53E-03	6.45E+02
2.58E-03	6.42E+02
2.58E-03	1.07E+03
2.63E-03	1.25E+03
2.69E-03	1.46E+03
2.69E-03	1.82E+03
3.00E-03	1.45E+03
3.18E-03	1.37E+03
3.18E-03	1.58E+03
3.43E-03	1.32E+03
3.70E-03	1.10E+03
3.70E-03	1.16E+03
3.85E-03	1.06E+03
4.00E-03	9.61E+02
4.00E-03	1.00E+03
4.00E-03	9.16E+02
5.00E-03	5.34E+02
6.00E-03	3.41E+02
8.00E-03	1.67E+02
9.66E-03	1.04E+02
9.66E-03	1.05E+02
1.00E-02	9.65E+01
1.34E-02	4.94E+01
1.34E-02	1.18E+02
1.50E-02	8.10E+01
1.57E-02	7.83E+01

1.57E-02	1.08E+02
1.61E-02	1.02E+02
1.64E-02	9.73E+01
1.64E-02	1.12E+02
2.00E-02	6.25E+01
3.00E-02	2.19E+01
4.00E-02	1.04E+01
5.00E-02	5.85E+00
6.00E-02	3.67E+00
8.00E-02	1.79E+00
9.05E-02	1.42E+00
9.05E-02	5.57E+00
1.00E-01	4.03E+00
1.50E-01	1.49E+00
2.00E-01	7.55E-01
3.00E-01	3.22E-01
4.00E-01	1.96E-01
5.00E-01	1.42E-01
6.00E-01	1.14E-01
8.00E-01	8.52E-02
1.00E+00	7.03E-02
1.02E+00	6.91E-02
1.25E+00	5.95E-02
1.50E+00	5.32E-02
2.00E+00	4.66E-02
2.04E+00	4.62E-02
3.00E+00	4.12E-02
4.00E+00	3.94E-02
5.00E+00	3.89E-02

Table S5. Mass attenuation coefficients (μ_m) of 800-phr-Pb/NR composites.

**Photon energy (MeV)**	μ_m **(cm²/g)**
1.00E-03	4.69E+03
1.01E-03	4.60E+03
1.02E-03	4.51E+03
1.02E-03	4.52E+03
1.03E-03	4.42E+03
1.04E-03	4.33E+03
1.04E-03	4.34E+03
1.12E-03	3.79E+03
1.19E-03	3.31E+03
1.19E-03	3.32E+03
1.50E-03	2.10E+03
2.00E-03	1.13E+03
2.47E-03	7.07E+02
2.47E-03	7.16E+02
2.53E-03	7.12E+02

2.58E-03	7.08E+02
2.58E-03	1.21E+03
2.63E-03	1.41E+03
2.69E-03	1.66E+03
2.69E-03	2.08E+03
3.00E-03	1.67E+03
3.18E-03	1.57E+03
3.18E-03	1.82E+03
3.43E-03	1.52E+03
3.70E-03	1.26E+03
3.70E-03	1.34E+03
3.85E-03	1.22E+03
4.00E-03	1.11E+03
4.00E-03	1.16E+03
4.00E-03	1.06E+03
5.00E-03	6.16E+02
6.00E-03	3.94E+02
8.00E-03	1.93E+02
9.66E-03	1.20E+02
9.66E-03	1.21E+02
1.00E-02	1.11E+02
1.34E-02	5.68E+01
1.34E-02	1.36E+02
1.50E-02	9.38E+01
1.57E-02	9.06E+01
1.57E-02	1.25E+02
1.61E-02	1.19E+02
1.64E-02	1.13E+02
1.64E-02	1.30E+02
2.00E-02	7.25E+01
3.00E-02	2.54E+01
4.00E-02	1.21E+01
5.00E-02	6.77E+00
6.00E-02	4.23E+00
8.00E-02	2.05E+00
9.05E-02	1.63E+00
9.05E-02	6.45E+00
1.00E-01	4.67E+00
1.50E-01	1.71E+00
2.00E-01	8.57E-01
3.00E-01	3.56E-01
4.00E-01	2.11E-01
5.00E-01	1.50E-01
6.00E-01	1.19E-01
8.00E-01	8.67E-02
1.00E+00	7.06E-02
1.02E+00	6.93E-02
1.25E+00	5.92E-02

1.50E+00	5.28E-02
2.00E+00	4.64E-02
2.04E+00	4.60E-02
3.00E+00	4.17E-02
4.00E+00	4.05E-02
5.00E+00	4.05E-02

Table S6. Mass attenuation coefficients (μ_m) of 400-phr-Bi_2S_3/NR composites.

Photon energy (MeV)	μ_m (cm²/g)
1.00E-03	4.08E+03
1.01E-03	4.00E+03
1.02E-03	3.92E+03
1.02E-03	3.93E+03
1.03E-03	3.85E+03
1.04E-03	3.77E+03
1.04E-03	3.78E+03
1.12E-03	3.28E+03
1.19E-03	2.85E+03
1.19E-03	2.86E+03
1.50E-03	1.77E+03
2.00E-03	9.34E+02
2.47E-03	5.76E+02
2.47E-03	8.40E+02
2.53E-03	8.02E+02
2.58E-03	7.64E+02
2.58E-03	1.35E+03
2.63E-03	1.36E+03
2.69E-03	1.37E+03
2.69E-03	1.79E+03
3.00E-03	1.42E+03
3.18E-03	1.23E+03
3.18E-03	1.39E+03
3.43E-03	1.15E+03
3.70E-03	9.53E+02
3.70E-03	1.00E+03
3.85E-03	9.11E+02
4.00E-03	8.28E+02
4.00E-03	8.60E+02
4.00E-03	8.59E+02
5.00E-03	4.98E+02
6.00E-03	3.17E+02
8.00E-03	1.54E+02
9.66E-03	9.54E+01
9.66E-03	9.70E+01
1.00E-02	8.88E+01
1.34E-02	4.20E+01
1.34E-02	9.52E+01

1.50E-02	7.07E+01
1.57E-02	6.26E+01
1.57E-02	8.53E+01
1.61E-02	8.11E+01
1.64E-02	7.72E+01
1.64E-02	8.86E+01
2.00E-02	5.36E+01
3.00E-02	1.89E+01
4.00E-02	8.96E+00
5.00E-02	5.05E+00
6.00E-02	3.17E+00
8.00E-02	1.56E+00
9.05E-02	1.16E+00
9.05E-02	4.39E+00
1.00E-01	3.42E+00
1.50E-01	1.28E+00
2.00E-01	6.58E-01
3.00E-01	2.90E-01
4.00E-01	1.82E-01
5.00E-01	1.35E-01
6.00E-01	1.10E-01
8.00E-01	8.38E-02
1.00E+00	7.00E-02
1.02E+00	6.88E-02
1.25E+00	5.97E-02
1.50E+00	5.35E-02
2.00E+00	4.67E-02
2.04E+00	4.63E-02
3.00E+00	4.08E-02
4.00E+00	3.84E-02
5.00E+00	3.75E-02

Table S7. Mass attenuation coefficients (μ_m) of 800-phr-Bi_2S_3/NR composites.

**Photon energy (MeV)**	μ_m **(cm²/g)**
1.00E-03	4.41E+03
1.01E-03	4.33E+03
1.02E-03	4.24E+03
1.02E-03	4.25E+03
1.03E-03	4.16E+03
1.04E-03	4.07E+03
1.04E-03	4.08E+03
1.12E-03	3.55E+03
1.19E-03	3.09E+03
1.19E-03	3.09E+03
1.50E-03	1.93E+03
2.00E-03	1.03E+03
2.47E-03	6.40E+02

2.47E-03	9.39E+02
2.53E-03	8.96E+02
2.58E-03	8.55E+02
2.58E-03	1.54E+03
2.63E-03	1.55E+03
2.69E-03	1.56E+03
2.69E-03	2.06E+03
3.00E-03	1.63E+03
3.18E-03	1.41E+03
3.18E-03	1.59E+03
3.43E-03	1.32E+03
3.70E-03	1.10E+03
3.70E-03	1.15E+03
3.85E-03	1.05E+03
4.00E-03	9.54E+02
4.00E-03	9.91E+02
4.00E-03	9.90E+02
5.00E-03	5.75E+02
6.00E-03	3.66E+02
8.00E-03	1.78E+02
9.66E-03	1.10E+02
9.66E-03	1.11E+02
1.00E-02	1.02E+02
1.34E-02	4.82E+01
1.34E-02	1.10E+02
1.50E-02	8.18E+01
1.57E-02	7.24E+01
1.57E-02	9.88E+01
1.61E-02	9.40E+01
1.64E-02	8.94E+01
1.64E-02	1.03E+02
2.00E-02	6.21E+01
3.00E-02	2.18E+01
4.00E-02	1.04E+01
5.00E-02	5.83E+00
6.00E-02	3.66E+00
8.00E-02	1.79E+00
9.05E-02	1.33E+00
9.05E-02	5.08E+00
1.00E-01	3.96E+00
1.50E-01	1.46E+00
2.00E-01	7.44E-01
3.00E-01	3.19E-01
4.00E-01	1.95E-01
5.00E-01	1.42E-01
6.00E-01	1.14E-01
8.00E-01	8.50E-02
1.00E+00	7.02E-02

1.02E+00	6.90E-02
1.25E+00	5.94E-02
1.50E+00	5.32E-02
2.00E+00	4.65E-02
2.04E+00	4.62E-02
3.00E+00	4.12E-02
4.00E+00	3.94E-02
5.00E+00	3.89E-02

Table S8. Mass attenuation coefficients (μ_m) of wood/NR composites.

**Photon energy (MeV)**	**μ_m (cm²/g)**
1.00E-03	2.19E+03
1.01E-03	2.13E+03
1.02E-03	2.07E+03
1.02E-03	2.12E+03
1.03E-03	2.08E+03
1.04E-03	2.06E+03
1.04E-03	2.10E+03
1.12E-03	1.76E+03
1.19E-03	1.48E+03
1.19E-03	1.50E+03
1.50E-03	7.99E+02
2.00E-03	3.54E+02
2.47E-03	1.92E+02
2.47E-03	2.42E+02
3.00E-03	1.42E+02
4.00E-03	6.24E+01
5.00E-03	3.28E+01
6.00E-03	1.93E+01
8.00E-03	8.36E+00
9.66E-03	4.86E+00
9.66E-03	9.82E+00
1.00E-02	8.97E+00
1.50E-02	3.06E+00
2.00E-02	1.47E+00
3.00E-02	5.90E-01
4.00E-02	3.60E-01
5.00E-02	2.72E-01
6.00E-02	2.30E-01
8.00E-02	1.91E-01
1.00E-01	1.72E-01
1.50E-01	1.48E-01
2.00E-01	1.33E-01
3.00E-01	1.15E-01
4.00E-01	1.03E-01
5.00E-01	9.38E-02
6.00E-01	8.67E-02

8.00E-01	7.61E-02
1.00E+00	6.84E-02
1.02E+00	6.77E-02
1.25E+00	6.12E-02
1.50E+00	5.57E-02
2.00E+00	4.78E-02
2.04E+00	4.72E-02
3.00E+00	3.83E-02
4.00E+00	3.28E-02
5.00E+00	2.92E-02

Table S9. Mass attenuation coefficients (μ_m) of 400-phr-Bi_2O_3/wood/NR composites.

Photon energy (MeV)	μ_m (cm^2/g)
1.00E-03	4.38E+03
1.01E-03	4.29E+03
1.02E-03	4.20E+03
1.02E-03	4.22E+03
1.03E-03	4.13E+03
1.04E-03	4.04E+03
1.04E-03	4.06E+03
1.12E-03	3.52E+03
1.19E-03	3.05E+03
1.19E-03	3.06E+03
1.50E-03	1.89E+03
2.00E-03	9.96E+02
2.47E-03	6.13E+02
2.47E-03	6.28E+02
2.53E-03	5.99E+02
2.58E-03	5.70E+02
2.58E-03	1.20E+03
2.63E-03	1.22E+03
2.69E-03	1.24E+03
2.69E-03	1.69E+03
3.00E-03	1.34E+03
3.18E-03	1.15E+03
3.18E-03	1.32E+03
3.43E-03	1.10E+03
3.70E-03	9.12E+02
3.70E-03	9.64E+02
3.85E-03	8.77E+02
4.00E-03	7.99E+02
4.00E-03	8.32E+02
4.00E-03	8.32E+02
5.00E-03	4.85E+02
6.00E-03	3.10E+02
8.00E-03	1.51E+02
9.66E-03	9.42E+01

9.66E-03	9.57E+01
1.00E-02	8.77E+01
1.34E-02	4.18E+01
1.34E-02	9.84E+01
1.50E-02	7.32E+01
1.57E-02	6.48E+01
1.57E-02	8.90E+01
1.61E-02	8.47E+01
1.64E-02	8.06E+01
1.64E-02	9.27E+01
2.00E-02	5.62E+01
3.00E-02	1.98E+01
4.00E-02	9.42E+00
5.00E-02	5.31E+00
6.00E-02	3.34E+00
8.00E-02	1.64E+00
9.05E-02	1.22E+00
9.05E-02	4.65E+00
1.00E-01	3.63E+00
1.50E-01	1.35E+00
2.00E-01	6.92E-01
3.00E-01	3.02E-01
4.00E-01	1.87E-01
5.00E-01	1.38E-01
6.00E-01	1.12E-01
8.00E-01	8.46E-02
1.00E+00	7.04E-02
1.02E+00	6.92E-02
1.25E+00	5.99E-02
1.50E+00	5.36E-02
2.00E+00	4.68E-02
2.04E+00	4.64E-02
3.00E+00	4.09E-02
4.00E+00	3.86E-02
5.00E+00	3.78E-02

Table S10. Mass attenuation coefficients (μ_m) of 800-phr-Bi_2O_3/wood/NR composites.

**Photon energy (MeV)**	μ_m **(cm^2/g)**
1.00E-03	4.78E+03
1.01E-03	4.68E+03
1.02E-03	4.59E+03
1.02E-03	4.60E+03
1.03E-03	4.50E+03
1.04E-03	4.40E+03
1.04E-03	4.41E+03
1.12E-03	3.84E+03
1.19E-03	3.34E+03

1.19E-03	3.34E+03
1.50E-03	2.09E+03
2.00E-03	1.11E+03
2.47E-03	6.89E+02
2.47E-03	6.99E+02
2.53E-03	6.66E+02
2.58E-03	6.35E+02
2.58E-03	1.38E+03
2.63E-03	1.40E+03
2.69E-03	1.42E+03
2.69E-03	1.96E+03
3.00E-03	1.55E+03
3.18E-03	1.34E+03
3.18E-03	1.54E+03
3.43E-03	1.28E+03
3.70E-03	1.06E+03
3.70E-03	1.12E+03
3.85E-03	1.02E+03
4.00E-03	9.32E+02
4.00E-03	9.72E+02
4.00E-03	9.71E+02
5.00E-03	5.67E+02
6.00E-03	3.62E+02
8.00E-03	1.77E+02
9.66E-03	1.10E+02
9.66E-03	1.11E+02
1.00E-02	1.02E+02
1.34E-02	4.86E+01
1.34E-02	1.16E+02
1.50E-02	8.59E+01
1.57E-02	7.60E+01
1.57E-02	1.05E+02
1.61E-02	9.96E+01
1.64E-02	9.47E+01
1.64E-02	1.09E+02
2.00E-02	6.61E+01
3.00E-02	2.33E+01
4.00E-02	1.11E+01
5.00E-02	6.22E+00
6.00E-02	3.90E+00
8.00E-02	1.90E+00
9.05E-02	1.41E+00
9.05E-02	5.47E+00
1.00E-01	4.26E+00
1.50E-01	1.57E+00
2.00E-01	7.93E-01
3.00E-01	3.36E-01
4.00E-01	2.02E-01

5.00E-01	1.46E-01
6.00E-01	1.16E-01
8.00E-01	8.61E-02
1.00E+00	7.08E-02
1.02E+00	6.95E-02
1.25E+00	5.96E-02
1.50E+00	5.33E-02
2.00E+00	4.66E-02
2.04E+00	4.62E-02
3.00E+00	4.14E-02
4.00E+00	3.97E-02
5.00E+00	3.93E-02

Table S11. Mass attenuation coefficients (μ_m) of 400-phr-Pb/wood/NR composites.

Photon energy (MeV)	μ_m (cm^2/g)
1.00E-03	4.28E+03
1.01E-03	4.20E+03
1.02E-03	4.11E+03
1.02E-03	4.13E+03
1.03E-03	4.04E+03
1.04E-03	3.96E+03
1.04E-03	3.97E+03
1.12E-03	3.46E+03
1.19E-03	3.01E+03
1.19E-03	3.01E+03
1.50E-03	1.88E+03
2.00E-03	9.99E+02
2.47E-03	6.20E+02
2.47E-03	6.35E+02
2.53E-03	6.32E+02
2.58E-03	6.28E+02
2.58E-03	1.04E+03
2.63E-03	1.21E+03
2.69E-03	1.41E+03
2.69E-03	1.76E+03
3.00E-03	1.41E+03
3.18E-03	1.33E+03
3.18E-03	1.53E+03
3.43E-03	1.28E+03
3.70E-03	1.06E+03
3.70E-03	1.13E+03
3.85E-03	1.02E+03
4.00E-03	9.30E+02
4.00E-03	9.69E+02
4.00E-03	8.86E+02
5.00E-03	5.16E+02

6.00E-03	3.30E+02
8.00E-03	1.61E+02
9.66E-03	1.00E+02
9.66E-03	1.02E+02
1.00E-02	9.33E+01
1.34E-02	4.78E+01
1.34E-02	1.14E+02
1.50E-02	7.83E+01
1.57E-02	7.56E+01
1.57E-02	1.04E+02
1.61E-02	9.87E+01
1.64E-02	9.39E+01
1.64E-02	1.08E+02
2.00E-02	6.03E+01
3.00E-02	2.12E+01
4.00E-02	1.01E+01
5.00E-02	5.66E+00
6.00E-02	3.55E+00
8.00E-02	1.74E+00
9.05E-02	1.38E+00
9.05E-02	5.38E+00
1.00E-01	3.90E+00
1.50E-01	1.44E+00
2.00E-01	7.33E-01
3.00E-01	3.15E-01
4.00E-01	1.93E-01
5.00E-01	1.41E-01
6.00E-01	1.13E-01
8.00E-01	8.48E-02
1.00E+00	7.02E-02
1.02E+00	6.90E-02
1.25E+00	5.95E-02
1.50E+00	5.33E-02
2.00E+00	4.66E-02
2.04E+00	4.62E-02
3.00E+00	4.11E-02
4.00E+00	3.92E-02
5.00E+00	3.86E-02

Table S12. Mass attenuation coefficients (μ_m) of 800-phr-Pb/wood/NR composites.

Photon energy (MeV)	μ_m (cm^2/g)
1.00E-03	4.66E+03
1.01E-03	4.57E+03
1.02E-03	4.48E+03
1.02E-03	4.49E+03
1.03E-03	4.39E+03
1.04E-03	4.30E+03

1.04E-03	4.31E+03
1.12E-03	3.76E+03
1.19E-03	3.28E+03
1.19E-03	3.29E+03
1.50E-03	2.07E+03
2.00E-03	1.12E+03
2.47E-03	6.97E+02
2.47E-03	7.07E+02
2.53E-03	7.03E+02
2.58E-03	6.99E+02
2.58E-03	1.19E+03
2.63E-03	1.39E+03
2.69E-03	1.63E+03
2.69E-03	2.04E+03
3.00E-03	1.63E+03
3.18E-03	1.54E+03
3.18E-03	1.78E+03
3.43E-03	1.49E+03
3.70E-03	1.24E+03
3.70E-03	1.31E+03
3.85E-03	1.19E+03
4.00E-03	1.09E+03
4.00E-03	1.13E+03
4.00E-03	1.04E+03
5.00E-03	6.04E+02
6.00E-03	3.86E+02
8.00E-03	1.89E+02
9.66E-03	1.18E+02
9.66E-03	1.18E+02
1.00E-02	1.09E+02
1.34E-02	5.57E+01
1.34E-02	1.34E+02
1.50E-02	9.19E+01
1.57E-02	8.88E+01
1.57E-02	1.22E+02
1.61E-02	1.16E+02
1.64E-02	1.11E+02
1.64E-02	1.27E+02
2.00E-02	7.10E+01
3.00E-02	2.49E+01
4.00E-02	1.18E+01
5.00E-02	6.63E+00
6.00E-02	4.15E+00
8.00E-02	2.02E+00
9.05E-02	1.60E+00
9.05E-02	6.32E+00
1.00E-01	4.57E+00
1.50E-01	1.68E+00

2.00E-01	8.42E-01
3.00E-01	3.51E-01
4.00E-01	2.09E-01
5.00E-01	1.49E-01
6.00E-01	1.18E-01
8.00E-01	8.64E-02
1.00E+00	7.05E-02
1.02E+00	6.93E-02
1.25E+00	5.92E-02
1.50E+00	5.29E-02
2.00E+00	4.64E-02
2.04E+00	4.60E-02
3.00E+00	4.16E-02
4.00E+00	4.03E-02
5.00E+00	4.03E-02

Table S13. Mass attenuation coefficients (μ_m) of 400-phr-Bi_2S_3/wood/NR composites.

**Photon energy (MeV)**	**μ_m (cm^2/g)**
1.00E-03	4.05E+03
1.01E-03	3.97E+03
1.02E-03	3.89E+03
1.02E-03	3.90E+03
1.03E-03	3.82E+03
1.04E-03	3.74E+03
1.04E-03	3.75E+03
1.12E-03	3.25E+03
1.19E-03	2.82E+03
1.19E-03	2.83E+03
1.50E-03	1.74E+03
2.00E-03	9.18E+02
2.47E-03	5.65E+02
2.47E-03	8.20E+02
2.53E-03	7.82E+02
2.58E-03	7.46E+02
2.58E-03	1.32E+03
2.63E-03	1.32E+03
2.69E-03	1.33E+03
2.69E-03	1.74E+03
3.00E-03	1.37E+03
3.18E-03	1.19E+03
3.18E-03	1.34E+03
3.43E-03	1.11E+03
3.70E-03	9.23E+02
3.70E-03	9.70E+02
3.85E-03	8.82E+02
4.00E-03	8.02E+02
4.00E-03	8.32E+02

4.00E-03	8.32E+02
5.00E-03	4.82E+02
6.00E-03	3.07E+02
8.00E-03	1.49E+02
9.66E-03	9.22E+01
9.66E-03	9.38E+01
1.00E-02	8.59E+01
1.34E-02	4.06E+01
1.34E-02	9.19E+01
1.50E-02	6.83E+01
1.57E-02	6.04E+01
1.57E-02	8.24E+01
1.61E-02	7.84E+01
1.64E-02	7.45E+01
1.64E-02	8.55E+01
2.00E-02	5.18E+01
3.00E-02	1.82E+01
4.00E-02	8.66E+00
5.00E-02	4.88E+00
6.00E-02	3.07E+00
8.00E-02	1.51E+00
9.05E-02	1.13E+00
9.05E-02	4.24E+00
1.00E-01	3.31E+00
1.50E-01	1.24E+00
2.00E-01	6.40E-01
3.00E-01	2.84E-01
4.00E-01	1.79E-01
5.00E-01	1.33E-01
6.00E-01	1.09E-01
8.00E-01	8.35E-02
1.00E+00	6.99E-02
1.02E+00	6.88E-02
1.25E+00	5.97E-02
1.50E+00	5.36E-02
2.00E+00	4.67E-02
2.04E+00	4.63E-02
3.00E+00	4.07E-02
4.00E+00	3.82E-02
5.00E+00	3.72E-02

Table S14. Mass attenuation coefficients (μ_m) of 800-phr-Bi_2S_3/wood/NR composites.

Photon energy (MeV)	μ_m (cm^2/g)
1.00E-03	4.39E+03
1.01E-03	4.30E+03
1.02E-03	4.22E+03
1.02E-03	4.22E+03

1.03E-03	4.13E+03
1.04E-03	4.04E+03
1.04E-03	4.05E+03
1.12E-03	3.52E+03
1.19E-03	3.07E+03
1.19E-03	3.07E+03
1.50E-03	1.92E+03
2.00E-03	1.02E+03
2.47E-03	6.32E+02
2.47E-03	9.25E+02
2.53E-03	8.83E+02
2.58E-03	8.42E+02
2.58E-03	1.51E+03
2.63E-03	1.52E+03
2.69E-03	1.54E+03
2.69E-03	2.02E+03
3.00E-03	1.60E+03
3.18E-03	1.38E+03
3.18E-03	1.56E+03
3.43E-03	1.30E+03
3.70E-03	1.08E+03
3.70E-03	1.13E+03
3.85E-03	1.03E+03
4.00E-03	9.36E+02
4.00E-03	9.72E+02
4.00E-03	9.71E+02
5.00E-03	5.64E+02
6.00E-03	3.59E+02
8.00E-03	1.74E+02
9.66E-03	1.08E+02
9.66E-03	1.09E+02
1.00E-02	9.98E+01
1.34E-02	4.72E+01
1.34E-02	1.08E+02
1.50E-02	8.01E+01
1.57E-02	7.09E+01
1.57E-02	9.68E+01
1.61E-02	9.21E+01
1.64E-02	8.76E+01
1.64E-02	1.01E+02
2.00E-02	6.09E+01
3.00E-02	2.14E+01
4.00E-02	1.02E+01
5.00E-02	5.72E+00
6.00E-02	3.59E+00
8.00E-02	1.75E+00
9.05E-02	1.30E+00
9.05E-02	4.98E+00

1.00E-01	3.88E+00
1.50E-01	1.44E+00
2.00E-01	7.32E-01
3.00E-01	3.15E-01
4.00E-01	1.93E-01
5.00E-01	1.41E-01
6.00E-01	1.13E-01
8.00E-01	8.48E-02
1.00E+00	7.02E-02
1.02E+00	6.90E-02
1.25E+00	5.94E-02
1.50E+00	5.32E-02
2.00E+00	4.66E-02
2.04E+00	4.62E-02
3.00E+00	4.11E-02
4.00E+00	3.92E-02
5.00E+00	3.87E-02
